# Marine Compounds Selectively Induce Apoptosis in Female Reproductive Cancer Cells but Not in Primary-Derived Human Reproductive Granulosa Cells

**DOI:** 10.3390/md10010064

**Published:** 2012-01-10

**Authors:** Vicki Edwards, Kirsten Benkendorff, Fiona Young

**Affiliations:** 1 Department of Medical Biotechnology, School of Medicine, Flinders University, GPO Box 2100, Adelaide, S.A. 5001, Australia; Email: edwa0223@flinders.edu.au (V.E.); Fiona.young@flinders.edu.au (F.Y.); 2 School of Biological Sciences, Flinders University, GPO Box 2100, Adelaide, S.A. 5001, Australia; 3 School of Environmental Sciences and Management, Southern Cross University, PO Box 157, Lismore, NSW 2480, Australia

**Keywords:** gynaecological cancers, brominated indoles, marine mollusc, apoptosis

## Abstract

Anticancer properties of tyrindoleninone and 6-bromoisatin from *Dicathais orbita* were tested against physiologically normal primary human granulosa cells (HGC) and reproductive cancer cell lines. Tyrindoleninone reduced cancer cell viability with IC_50_ values of 39 µM (KGN; a tumour-derived granulosa cell line), 39 μM (JAr), and 156 μM (OVCAR-3), compared to 3516 μM in HGC. Apoptosis in HGC’s occurred after 4 h at 391 µM tyrindoleninone compared to 20 µM in KGN cells. Differences in apoptosis between HGC and KGN cells were confirmed by TUNEL, with 66 and 31% apoptotic nuclei at 4 h in KGN and HGC, respectively. These marine compounds therefore have potential for development as treatments for female reproductive cancers.

## 1. Introduction

Gynaecological cancers of the ovary, cervix and endometrial are less common than either lung or breast cancer in women, but the mortality rates are higher due to late diagnosis [1,2]. Ovarian cancer presents very few symptoms, yet is a rapidly growing aggressive cancer [3], with the highest incidence of mortality of all the gynaecological cancers, and a 5-year survival rate of 26% [4]. While the incidence of cervical cancer is decreasing in developed countries, it is still the second most common gynaecological cancer world-wide, with over half a million cases diagnosed each year [5]. Because women often present late with gynaecological cancers, treatments are often aggressive but fatalities still occur from relapse of the disease [6].

Nature has been an important source of novel anti-cancer drug leads over the past 25 years [7] with increasing numbers of new compounds sourced from the marine environment [8,9,10,11,12,13]. A recent economic analysis has estimated the value of anti-cancer drugs of marine origin at US $563 billion to 5.69 trillion pending discovery, with up to 214 new compounds predicted to reach the market in the future [14]. The novel and diverse structures of marine compounds makes them a preferred source for new drug candidates [10,15] especially for multi-drug resistant carcinomas, such as ovarian cancer [6,16]. The drug Trabectedin (Yondelis^®^; PharmaMar), isolated from the marine tunicate, *Ecteinascidia turbinata*, has a unique mode of action; it inhibits several transcription factors *in vitro* and *in vivo* [17,18] and has now been approved for the treatment of platinum-sensitive ovarian cancer and tumour soft tissue sarcoma [19]. A range of bioactive compounds with anticancer properties have also been isolated from molluscs [10,15,20,21]. Dolastatin-10, and 15, derived from the shell-less mollusc *Dolabella auricularia* [22], were reported to have anti-tumour activity against breast and liver cancer in phase I clinical trials [23]. In phase II trials, however Dolastatin-10 had minimal activity against breast and platinum-sensitive ovarian cancer [24,25]. Another bioactive compound Kahalalide F, isolated from the marine mollusc, *Elysia rufescens* [26], has shown anti-tumour activity in breast, hepatoma, melanoma and pancreatic carcinomas in phase I clinical trials [9,27]. A synthetic derivative PM02734 of Kahalalide F, induces apoptosis in lung cancer cell lines (H322 and A549), *in vitro* and *in vivo* and is currently undergoing phase II clinical trials [28]. The compound, ES-285–HCl originally isolated from the clam *Spisula polynyma*, has shown selective anticancer properties against several cancer cell lines *in vitro* and against solid hepatocellular, prostate and renal tumours *in vivo* [29].

The indole derivatives tyrindoleninone, tyrindolinone, 6-bromoisatin and 6,6′-dibromoindirubin, from the Muricidae family of marine gastropods, also have anti-cancer properties [30,31,32]. *In vitro*, these indole compounds inhibited cell growth in solid tumour cell lines from the colon and breast, and induced apoptosis and necrosis in T-cell lymphoma cells [30]. Tyrindoleninone in particular, is cytotoxic against the human lymphoma cell lines, U937 and Jurkat (IC_50_ = 3.9 μM; 1 μg/mL) in comparison to the untransformed, human, mononuclear cells (MNC) (IC_50_ = 195 μM; 50 μg/mL) *in vitro* [30,31]. *In vivo* studies in a rodent model for the prevention of colon cancer have also shown that crude extracts from the muricid, *Dicathais orbita* (Muricidae, Gastropoda) containing these compounds are pro-apoptotic in cells of the distal colon in response to administration of the genotoxic agent, azoxymethane [32]. 

The muricid family of whelks is the source of a homeopathic remedy “Murex purpurea”, recommended for the treatment of gynaecological disorders including cancer of the uterus [33,34], but the remedy had little or no effect on cell proliferation across a range of cancer cell lines *in vitro* [30]. Only trace amounts of 6-bromoisatin were detected in the homeopathic remedy used in this study [30]. However, indirubin, a related compound is the active ingredient in the traditional Chinese medicine, “Danggui Luhui Wan”, used for treating leukaemia [35] and induces apoptosis in prostate and lung cancer [36]. Isatins and analogues of isatin also have anti-proliferative and anti-cancer properties *in vitro* [31,37]. 5- and 7-bromoisatin act by inhibiting micro-tubular formation in cancer cell lines *in vitro* [37]. Vine *et al.* [31] demonstrated that a range of isatins including 5 and 7-dibromoisatin selectively promoted apoptosis in the lymphoma cell lines U937 and Jurkat, by the activation of effector caspases-3 and -7. 

The brominated indoles isolated from *Dicathais orbita* have not been tested for efficacy against aggressive gynaecological cancers. We therefore aimed to determine if compounds from *D. orbita* could selectively target human female reproductive cancer cell lines without causing significant cell death to primary-derived human reproductive cells. There are a wide range of human reproductive cancer cell lines available, including the KGN granulosa tumour cell line, established in 2001 from a 63-year old women with an invasive carcinoma [38], the OVCAR-3 cancer cell line, originally derived from an adenocarcinoma of the ovary [39], and the JAr choriocarcinoma cell line established from a trophoblastic tumour of the placenta [40]. Primary human reproductive granulosa cells (HGC) derived from women with normal reproductive physiology undergoing assisted reproductive technologies (ART) were used in this study as a direct comparison to the KGN granulosa tumour cell line. The cytotoxic mode of action in KGN and HGCs was examined by using membrane integrity assays (LDH release) to identify necrosis, and apoptosis was examined by measurement of DNA fragmentation (TUNEL) and enzyme activity (caspase-3/7). As no marine natural products appear to have been previously tested for cytotoxicity against human primary granulosa cells, this study also presents a new model for screening anticancer agents specifically for female reproductive cancers.

## 2. Results and Discussion

### 2.1. Compound Identification

**Figure 1:**
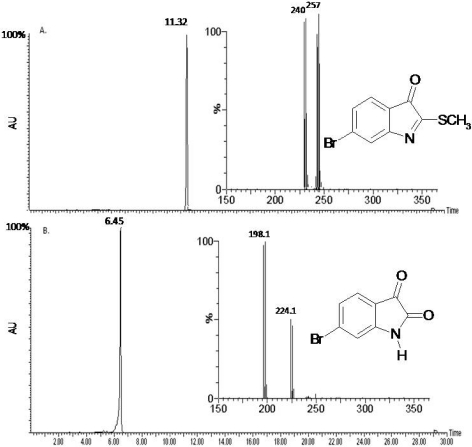
LC/MS analysis of the two purified fractions extracted from the egg capsules of *D. orbita*. (**A**) The chromatogram from the Diode array at 300 + 600 nm shows the retention times (*t*_R_) and relative peak of the main indole compound in the first fraction at *t*_R_ 11.32 min. This fraction was confirmed to be tyrindoleninone by the molecular mass (ESI/MS insert; *m*/*z* 255, 257). (**B**) The second purified fraction at *t*_R_ 6.45 min corresponded to that of 6-bromoisatin by its molecular weight (ESI/MS insert; *m*/*z* 224, 226).

The previously reported compounds, tyrindoleninone and 6-bromisatin from the marine whelk *D. orbita* [32,41], were readily isolated and purified from a crude egg capsule extract (3.726 g). LC/MS of the purified fractions identified one major compound in fraction one (0.120 g) at *t*_R_ 11.32 min which corresponded to the molecular mass of tyrindoleninone (ESI/MS insert *m*/*z* 255, 257 isotopes for bromine Br^79^, Br^81^; Figure 1A). The fragmented ion at *m*/*z* 240 correlated to the loss of a hydroxyl group (-H_2_O) from the tyrindoleninone compound. The second fraction (0.105 g) consisted of one major HPLC peak at *t*_R_ 6.45 min which was confirmed to be 6-bromoisatin by the molecular mass on the ESI/MS (insert; *m*/*z* 224, 226 for Br^79^, Br^81^; Figure 1B). The major fragment ion at *m*/*z* 198 is due to the loss of CO. 

### 2.2. Biological Activity of the *D. orbita* Compounds

#### 2.2.1. Cell Viability

Selective inhibition of cancer cell lines is a major advantage for anticancer agents, as non specific cytotoxicity in primary cells can cause undesirable side effects preventing FDA drug approval [10,30]. The two *D. orbita* fractions were tested for their cytotoxicity against three reproductive cancer cells lines and primary-derived human reproductive granulosa cells (HGC) using the crystal violet and MTT assay. Tyrindoleninone (fraction one) was more active against the reproductive cancer lines than the HGC and inhibited the metabolic function of the cells (MTT assay) before cell death occurred (Crystal violet assay; Table 1). Tyrindoleninone selectively reduced the viability of all three reproductive cancer cell lines KGN (IC_50_ = 39 µM MTT and 156 µM Crystal violet), JAr (IC_50_ = 39 µM MTT and 234 µM Crystal violet) and OVCAR-3 (IC_50_ = 156 µM MTT and 313 µM Crystal violet) at much lower concentrations than the corresponding primary cells (IC_50_ = 3516 µM MTT and >1953 µM Crystal violet) after just 4 h exposure (Table 1). After 24, 48 and 72 h treatment, tyrindoleninone was significantly more cytotoxic towards all three reproductive cancer cell lines, with IC_50_ values approximately 10-fold lower than for the HGC (Table 1). Benkendorff *et al.* [30] showed a 50% reduction in the MCF-7 breast, CaCo_2_ colon and U937 lymphoma carcinoma cell viability after 4 h exposure to 391 µM tyrindoleninone in an impure extract compared to untransformed mononuclear cells. Another study predicted a 50% reduction in lymphoma cell viability after 1 h exposure to 4 µM tyrindoleninone [31]. Furthermore, Vine *et al.* [31] identified that tyrindoleninone was much less cytotoxic to untransformed human mononuclear cells with IC_50_ = 195 µM after 1 h exposure. The different inhibitory concentrations in the various studies is likely to be due to the relative purity of tyrindoleninone in the extract tested, as well as innate differences in the susceptibility of different cell lines. Furthermore, Vine *et al.* [31] noted that a contaminating plasticizer in their study may have increased the solubility and availability of tyrindoleninone to the cells. Cell types vary with regards to their metabolic rate and cell culture conditions (such as cell density and passage numbers) and this can also influence the outcome of an assay [42].

**Table 1:** Cytotoxicity of tyrindoleninone and 6-bromoisatin to primary-derived human reproductive granulosa cells (HGC), KGN, JAr and OVCAR-3 cells determined by the crystal violet (CV) and the MTT assays, and results are shown as IC_50_ the concentration that inhibited 50% of the cells. The values are mean of three independent assays (*n* = 3). n/t: not tested; >: concentration is greater than; <: concentration is less than.

Cell Type	Time (h)	IC_50_ (µM)			
		Tyrindoleninone		6-Bromoisatin	
		CV	MTT	CV	MTT
HGC	4	>1953	3516	>2232	>4464
KGN		156	39	892	178
JAr		234	39	223	223
OVCAR-3		313	156	446	402
HGC	24	>1953	1563	>2232	>4464
KGN		156	78	223	446
JAr		195	117	178	446
OVCAR-3		352	234	268	446
HGC	48	n/t	1563	n/t	1785
KGN		n/t	20	n/t	22
JAr		n/t	78	n/t	446
OVCAR-3		n/t	31	n/t	89
HGC	72	n/t	1563	n/t	2232
KGN		n/t	<20	n/t	<22
JAr		n/t	n/t	n/t	n/t
OVCAR-3		n/t	<20	n/t	<22

6-Bromoisatin, while not as potent as tyrindoleninone, still significantly decreased cell numbers in all three reproductive cancer cell lines, whilst having only a minimal effect on HGC (Table 1). The JAr cell line was the most sensitive after shorter incubation periods, with cell numbers halved at 223 µM (Crystal violet and MTT assay) and 178 µM and 446 µM (Crystal violet and MTT assay) of 6-bromoisatin after 4 and 24 h exposures respectively (IC_50_; Table 1). In comparison, the HGC numbers were only reduced by >2232 µM 6-bromoisatin after 24 h exposure. After 48 and 72 h exposure to 6-bromoisatin the concentration that inhibited 50% of KGN and OVCAR-3 cell lines was much lower (22 µM 48 and 72 h KGN and 89 and 22 µM OVCAR-3) as determined by the MTT assay. Vine *et al.* [31] have demonstrated that a range of substituted isatins including 5 and 7-dibromoisatin are 10× more active against the lymphoma cell lines, U937 and Jurkat than 6-bromoisatin. Nevertheless, the results from this study and previous research on the anticancer properties of these marine compounds in particular tyrindoleninone, all support the specificity of these compounds towards rapidly dividing cancer cell lines over freshly isolated healthy human cells.

#### 2.2.2. Mode of Action Investigation: Apoptosis and Necrosis Assays

**Figure 2:**
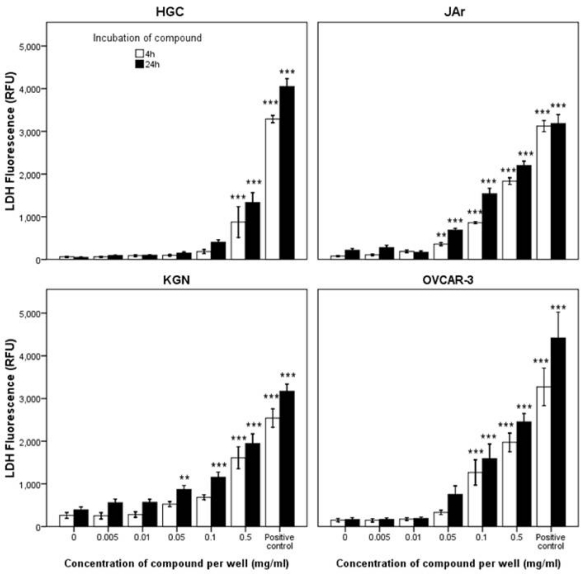
Effects of tyrindoleninone on LDH release in the primary-derived human reproductive granulosa (HGC), KGN, JAr and OVCAR-3 cells. After an initial 2 h (JAr) and 24 h (HGC, KGN and OVCAR-3) cell attachment period the cells (10,000 cells/well) were treated with tyrindoleninone for 4 and 24 h. LDH release was measured by fluorescence at 535_EX_/590_EM_. The results are mean for three separate repeat assays (*n* = 3; ±1 SEM). Univariate analysis of variance with contrast (K Matrix) were conducted to compare the effects of the concentration of tyrindoleninone on LDH release against the 1% DMSO control shown as 0 concentration at 4 and 24 h. The positive control represents lysis buffer (Promega). Significant difference between each treatment and the 1% DMSO control at 4 and 24 h are shown as *p* < 0.01 (**) and *p* < 0.001 (***).

**Figure 3:**
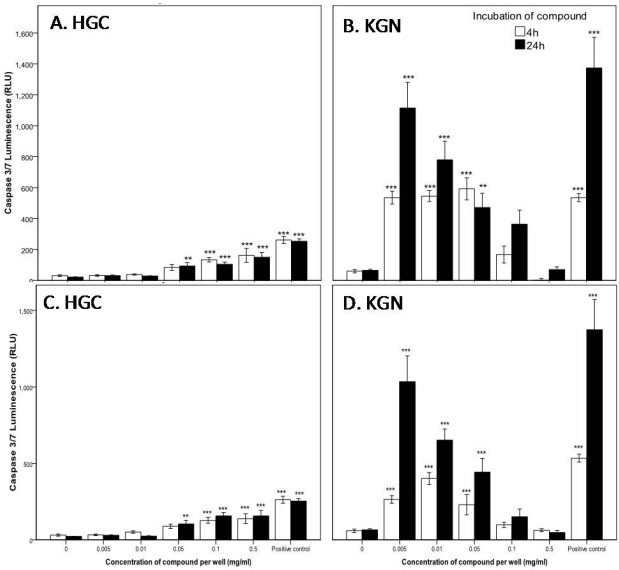
Up-regulation of caspase-3 and 7 in the primary-derived human reproductive granulosa (HGC) and KGN cells after incubation with tyrindoleninone (**A**, **B**) and 6-bromoisatin (**C**, **D**). After an initial 24 h cell attachment period cells (10,000 cells/well) were treated with tyrindoleninone and 6-bromoisatin for 4 and 24 h. Caspase-3 and -7 activity was measured at full light on an luminescence plate reader. The results are mean for three separate repeat assays (*n* = 3; ±1 SEM). The positive control represents exposure of cells to 1 µg/mL DNase I. Univariate analysis of variance with contrast (K Matrix) were conducted to compare the effects of each compound on caspase-3 and -7 activity against the 1% DMSO control shown as 0 concentration at 4 and 24 h. Significant difference between each treatment and the 1% DMSO control at 4 and 24 h are shown as *p* < 0.01 (**) and *p* < 0.001 (***).

Cell death by necrosis leads to the loss of cell membrane integrity and uncontrolled release of the cellular contents into the surrounding tissue causing inflammation [43] and as such is often considered a toxic process in comparison to apoptosis [44]. It therefore is an advantage to identify anticancer agents that specifically induce apoptosis as opposed to necrosis. After exposure of the granulosa cancer cells KGN, and primary HGC to tyrindoleninone and 6-bromoisatin, caspase-3 and -7 enzymes were activated at much lower concentrations than those required to cause disruption of membrane integrity, indicating cell death occurred predominately by apoptosis as opposed to necrosis. For example, a four hour exposure to 1953 µM (0.5 mg/mL) tyrindoleninone (Figure 2) was required to cause LDH release in both HGC and the corresponding KGN granulosa cell line, with more LDH being released by KGN (1612 ± 771) than primary HGC (876 ± 1084). This effect was more marked after a 24 h exposure, when lower concentrations (195 µM; 0.05 mg/mL) caused LDH release from KGN cells, as opposed to primary HGC (1953 µM; 0.5 mg/mL). In comparison, caspase-3 and -7 activity was noted in HGC after 4 h treatment with 391 µM (0.1 mg/mL) tyrindoleninone (*p* < 0.01; Figure 3A), whereas the corresponding granulosa cancer cell line, KGN, had significant increases in caspase-3 and -7 activity at 20 µM (0.005 mg/mL; *p* < 0.001; Figure 3B). In fact, there was greater capsase-3 and -7 activity detected overall by the KGN cells than in the corresponding primary HGC in the presence of tyrindoleninone. Furthermore, there was a dose dependant decrease in KGN caspase activity as the concentration of tyrindoleninone increased, whereas there was a dose dependent increase for HGC (Figure 3B). Finally, necrosis was only detected in the JAr and OVCAR-3 cells after 4 h exposure to 195 µM (0.05 mg/mL) and 391 µM (0.1 mg/mL) of tyrindoleninone respectively (Figure 2).

**Figure 4:**
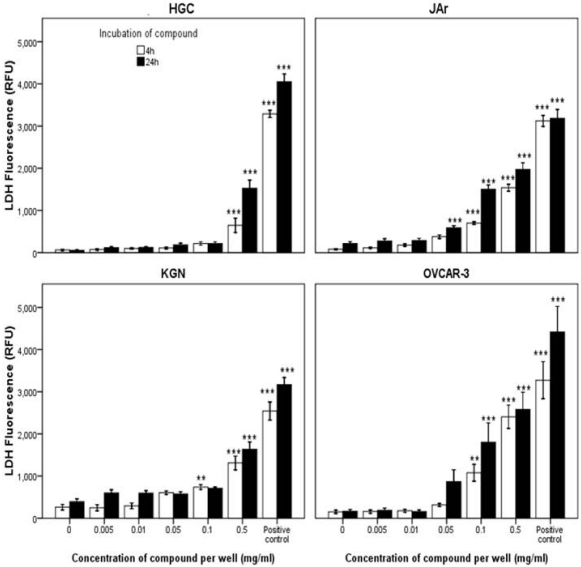
Effects of 6-bromoisatin on LDH release in the primary-derived human reproductive granulosa (HGC), KGN, JAr and OVCAR-3 cells. After an initial 2 (JAr) and 24 h (primary HGC, KGN and OVCAR-3) cell attachment period the cells (10,000 cells/well) were treated with 6-bromoisatin for 4 and 24 h. LDH release was measured by fluorescence at 535_EX_/590_EM_. The results are mean for three separate repeat assays (*n* = 3; ±1 SEM). Univariate analysis of variance with contrast (K Matrix) were conducted to compare the effects of the concentration of 6-bromoisatin on LDH release against the 1% DMSO control shown as 0 concentration at 4 and 24 h. The positive control represents a lysis buffer (Promega). Significant difference between each treatment and the 1% DMSO control at 4 and 24 h are shown as *p* < 0.01 (**) and *p* < 0.001 (***).

In a similar manner to tyrindoleninone, 6-bromoisatin significantly affected the reproductive cancer cell lines at lower concentrations than the HGC (Figure 4). Necrosis, as indicated by LDH release, was only significantly increased after 4 and 24 h exposure to 2232 µM (0.5 mg/mL) of 6-bromoisatin in HGC. In comparison, LDH release significantly increased after exposure to 446 µM (0.1 mg/mL) in the KGN, JAr and OVCAR-3 cells lines (Figure 4). Like tyrindoleninone, caspase-3 and -7 activity was only detected in HGC after 4 h treatment with 0.1 mg/mL 6-bromoisatin (*p* < 0.001; Figure 3C). In comparison KGN cell apoptotic activity was significantly higher after 4 h treatment with 22 µM (0.005 mg/mL) of 6-bromoisatin (*p* < 0.001; Figure 3D). When KGN cells were treated with 22–2232 µM (0.005–0.5 mg/mL) of 6-bromoisatin for 24 h, caspase-3 and 7 significantly decreased, in a dose-dependent manner (Figure 3D).

Other structurally similar indole and isatin compounds have also been shown to induce apoptosis by the activation of caspase-3 at low concentrations, in a range of cell lines *in vitro* [31,45,46]. For example 5,6,7-tribromoisatin activates caspase-3 and -7 at 8 µM (0.003 mg/mL) after 5 h in the Jurkat cell line [31]. Other research suggests that indoles and isatins inhibit cell proliferation and activate apoptosis by binding and inhibiting signalling of extracellular protein kinases (ERKs) [47]. As ERK signalling pathways, such as the ERK/MARK phosphatase pathway, are essential in cell proliferation and survival [48] it only follows that an inhibition of ERK would suppress growth and induce apoptosis in the cells.

#### 2.2.3. Confirmation of Apoptosis by TUNEL Staining

**Figure 5:**
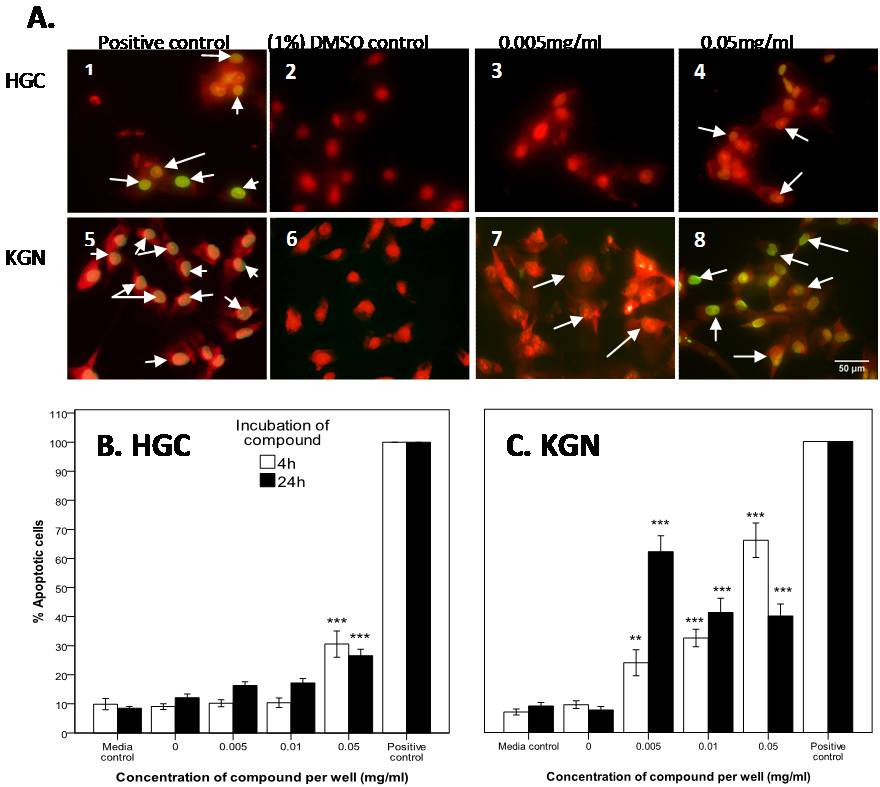
TUNEL staining of primary-derived reproductive human granulosa cells (HGC) (**A** 1–4) and KGN granulosa tumor cell line (**A** 5–8) after 4 h treatment with *D. orbita* fraction 1 containing tyrindoleninone. The percentage of apoptotic cells after HGC (**B**) and KGN cells (**C**) after treatment with tyrindoleninone for 4 h and 24 h. The positive control represents 1 µg/mL DNase I (C1 and 5), for comparison with the 1% DMSO control (C2 and 6), 0.005 mg/mL (C3 and 7) and 0.05 mg/mL (C4 and 8) of tyrindoleninone. Photomicrographs were taken of four random microscope fields, and the number of TUNEL positive nuclei were calculated as a fraction of the total number of PI stained nuclei in each image. The average of the four fields was then calculated and the results of (A) and (B) are the mean ± 1 SEM of 3 repeat assays (*n* = 3). Univariate analysis of variance with contrast (K Matrix) were conducted to determine the percentage of apoptotic cells from total cells induced by tyrindoleninone against the 1% DMSO control shown as 0 concentration at 4 and 24 h. Significant difference between each treatment and the 1% DMSO control at 4 and 24 h shown as *p* < 0.01 (**) and *p* < 0.001 (***). (C) Pictures represent overlaid apoptotic stain (green) and nuclear stain (red), and arrows represent DNA fragmentation after treatment for 4 h. Photographed on a Fluorescence Olympus AX70 Microscope at 400× magnification. Scale bar = 50 µM.

**Figure 6:**
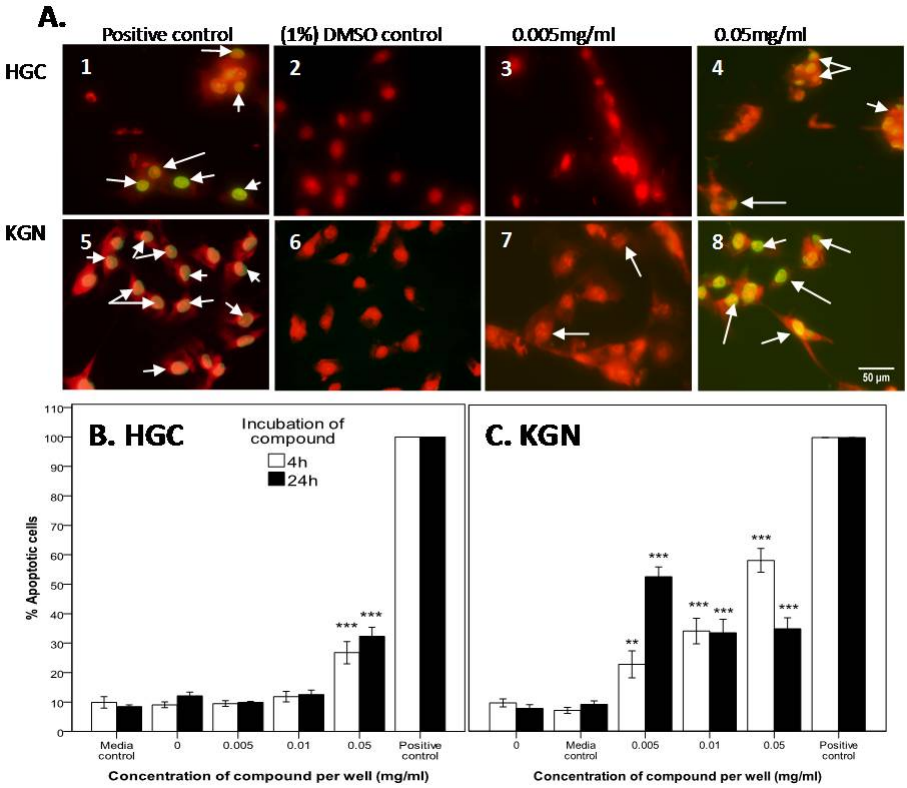
TUNEL staining of primary-derived reproductive human granulosa cells (HGC) (**A** 1–4) and KGN granulosa tumor cell line (**A** 5–8) after 4 h treatment with *D. orbita* fraction 2 containing 6-bromoisatin. The percentage of apoptotic cells after HGC (**B**) and KGN cells (**C**) after treatment with 6-bromoisatin for 4 h and 24 h. The positive control represents 1 µg/mL DNase I (C1 and 5), for compound with the 1% DMSO control (C2 and 6), 0.005 mg/mL (C3 and 7) and 0.05 mg/mL (C4 and 8) of 6-bromoisatin. Photo micrographs were taken of four random microscope fields, and the number of TUNEL positive nuclei were calculated as a fraction of the total number of PI stained nuclei in each image. The average of the four fields was then calculated and the results of (A) and (B) are the mean ± 1 SEM of 3 repeat assays (*n* = 3). Univariate analysis of variance with contrast (K Matrix) were conducted to determine the percentage of apoptotic cells from total cells induced by 6-bromoisatin against the 1% DMSO control shown as 0 concentration at 4 and 24 h. Significant difference between each treatment and the 1% DMSO control at 4 and 24 h shown as *p* < 0.01 (**) and *p* < 0.001 (***). (C) Pictures represent overlaid apoptotic stain (green) and nuclear stain (red), and arrows represent DNA fragmentation after treatment for 4 h. Photographed on a Fluorescence Olympus AX70 Microscope at 400× magnification. Scale bar = 50 µM.

To further assess whether the two *D. orbita* fractions containing the bioactive compounds tyrindoleninone and 6-bromoisatin induced cell death by apoptosis, the TUNEL assay was performed on the HGC and KGN cells. The DNase I treated HGC and KGN cells (positive controls) were 100% TUNEL stained (Figure 5 and Figure 6), whereas the negative controls (absence of terminal deoxynecleotide transferase) were TUNEL negative. After 4 and 24 h incubation, 9 and 12% of untreated (DMSO control) HGC, and 10 and 8% of KGN cells were TUNEL labelled (Figure 5 and Figure 6).

In the presence of 0.05 mg/mL tyrindoleninone after 4 h treatment, 31% of HGC (Figure 5A,C) and 66% of KGN cells were TUNEL positive (Figure 5B,C). After 24 h exposure to 20 µM (0.005 mg/mL) of tyrindoleninone only 16% of HGC were labelled, in comparison to 62% of KGN cells (*p* < 0.001; Figure 5A,B). DNA fragmentation after 4 h exposure to 10 tyrindoleninone in both HGC and KGN therefore occurred at a lower concentration (0.05 mg/mL) than that which caused LDH release (1953 µM or 0.5 mg/mL; Figure 2).

When HGC and KGN cells were treated with 6-bromoisatin at 223 µM (0.05 mg/mL) for 4 h, 27% of primary HGC were TUNEL positive (Figure 6A,C), whereas 58% of KGN cells were positive (*p* < 0.001; Figure 6B,C), and after 24 h of treatment with 6-bromoisatin (22 µM or 0.005 mg/mL), 10% of HGC were TUNEL labelled in comparison to a statistically significant increase in TUNEL positive KGN cells of 53% (*p* < 0.001; Figure 6A,B). In a similar manner to tyrindoleninone, when treated with 6-bromoisatin TUNEL fluorescence indicating DNA fragmentation was generally detected at lower concentrations than required to induce necrosis by LDH release (2232 µM or 0.5 mg/mL; Figure 4).

The consensus is that apoptosis can be activated via two main pathways, the extrinsic (cell surface activation) or intrinsic (mitochondrial activation) pathways [44]. Apoptosis in the KGN and COV434 human granulosa cancer cell lines treated with cisplatin was attributed to the extrinsic receptor pathway [49]. The extrinsic pathway via the Fas-Fas mediated ligand system was also implicated in KGN apoptosis when KGN cells exposed to interferon-γ underwent Fas-induced apoptosis [38]. Furthermore, the Fas-Fas pathway was involved in primary granulosa-luteal cell apoptosis [50]. TRAIL apoptosis via the intrinsic pathway has also been identified in primary granulosa and theca cells of the ovary, regulated by Bcl-212 proteins [51]. Therefore, it could be hypothesized that tyrindoleninone and 6-bromoisatin inhibited cell proliferation and activated apoptosis in the KGN granulosa cancer cell line via the TRAIL-induced or Fas-Fas pathways. Future studies, including investigation of initiator caspase 8 and 9, could help discriminate between the extrinsic and intrinsic pathway for the induction of apoptosis by these brominated indoles.

At higher concentrations of tyrindoleninone and 6-bromoisatin (≥0.05 mg/mL) a dose dependent increase in LDH release was observed in the KGN cells, HGC, JAr and OVCAR-3 cells. Our caspase-3/7 and TUNEL results suggest that tyrindoleninone and 6-bromoisatin activate cell death by apoptosis at low concentrations, but cause secondary necrosis or necrosis at higher concentrations [42,52]. In other words, at higher concentrations the apoptotic pathway was terminated and secondary necrosis may have been triggered. Benkendorff *et al.* [30] have shown that a crude extract (0.5 mg/mL) from the egg masses of *D. orbita* induced both apoptosis and necrosis in the T-cell lymphoma, Jurkat cell line, and necrosis alone in the HT29, solid colon cancer cell line after a 4 h exposure [30]. The authors of that study pointed out that cell death by apoptosis in lymphoma cell lines is congruent with published studies, whereas necrosis is more common in solid tumour cell lines [30].

Interestingly, *in vivo* studies have demonstrated that crude *D. orbita* extracts successfully induced apoptosis in cells of the distal colon in response to genotoxic damage [32]. High concentrations of the *D. orbita* extracts (1 mg/mL) induced a significantly greater rate of apoptosis than low concentrations (0.125 mg/mL) *in vivo* [32]. This is likely to be related to *in vivo* degradation and metabolism of the active compounds, thus requiring higher concentrations for oral delivery than is necessary to induce an effect *in vitro*. The induction of apoptosis at lower concentrations in our study compared to previous studies may also be related to testing the purified compounds rather than the crude extract in the apoptosis assays. Different concentrations of these muricid compounds possibly stimulate different cell death pathways in different cell lines and therefore in the KGN reproductive cells, apoptosis is being activated at low concentrations, whereas secondary necrosis or necrosis is activated at high concentrations.

## 3. Experimental Section

### 3.1. Extraction, Purification and Chemical Analysis

All chemicals used in this research were HPLC grade and purchased from Sigma-Aldrich unless otherwise stated. Bioactive compounds were extracted from *Dicathais orbita* egg masses that were collected from re-circulating tanks at Flinders University, South Australia. The egg capsules (285 g) were cut open and soaked in chloroform and methanol (v/v) for 24 h. The chloroform layer was then separated from the aqueous layer, filtered to remove tissue residue and evaporated to dryness under vacuum pressure (474 mbar; 40 °C) on a Buchi Rotary evaporator, yielding 3.726 g of a brown/red oil. This extract was subjected to liquid chromatography (Waters, Milford, USA; 2695 Separation Module and 2487 dual wavelength UV detector) coupled to an electrospray ionization mass spectrometer (ESI; Micromass Quattro micro™), with UV detection at wavelengths 300 and 600 nm, and data was acquired by the Masslynx Software as previously described [30] The brown/red oil was stored at −20 °C under N_2_ gas in amber vials until semi-purified. 

To facilitate the separation of the bioactive compounds tyrindoleninone and 6-bromoisatin, the crude samples extracted from the egg masses were semi-purified using flash silica chromatography under N_2_ pressure using a solvent system with increasing polarity of dichloromethane (DCM), 5% methanol in DCM and 10% methanol in DCM (Fraction 1: 0.120 g eluted from the column with DCM and Fraction 2: 0.105 g eluted from the column with 5% DCM). The collected fractions were dried on a Buchi Rotary evaporator and stored under N_2_ at −20 °C in amber vials prior to cell culture assays. LC/MS analysis was performed to identify the bioactive compounds in the two semi-purified fractions. 

For cell assays, the *D. orbita* fractions were prepared fresh on the day of the experiment by dissolving in dimethyl sulfoxide (DMSO; 100%) at 100× the final concentration. These were diluted to a range of working concentrations (0.001–0.5 mg/mL) in complete cell assay medium to give a final working concentration per well of 1% DMSO. All samples were filtered through a 0.22 μM (Sartorius) filter before use.

### 3.2. Cell Culture

#### 3.2.1. Isolation of Primary-Derived Human Granulosa Cells

Primary-derived human reproductive granulosa cells (HCG) were isolated from the follicular aspirates donated by women (*n* = 3) undergoing assisted reproductive technology (ART) at Flinders Medical Centre, Adelaide, South Australia under a protocol approved by the Flinders Clinical Research Ethics Committee (260/067). Granulosa cells were donated by women with normal reproductive physiology who were undergoing ART to treat male infertility. Granulosa cell isolation has been described previously [53]. Briefly, pooled aspirates for each woman were isolated by centrifugation at 107× *g* for 10 min, followed by two cycles of rinsing and then the cell suspension was re-suspended in Dulbecco’s modified Eagle’s HAMS F12 (DMEM-F12; GIBCO, Invitrogen Corporation) mediumsupplemented with 10% FBS (GIBCO Invitrogen Corporation), penicillin/streptomycin 5000 IU/mL and 5000 μg/mL respectively (Thermo Scientific), insulin (5 μg/mL), transferin apohuman (5 μg/mL), selenium sodium selenite (5 ng/mL) buffered with 1.2 g/L NaHCO_3_ (Pfizer). Granulosa cells were separated from the red blood cells using a lymphoprep (Ficoll-Paque TMPlus) column. The purified granulosa cells were rinsed and re-suspended in DMEM/F12 complete medium.

#### 3.2.2. Cell Line Culture

The KGN granulosa cell line [38] was maintained in the same DMEM/F12 complete media as the HCG. The JAr [40] and OVCAR-3 cell lines [39] obtained from the (Global Bioresource Centre™ American Tissue Culture Collection) were maintained in RPMI-1640 medium supplemented with 10 and 20% FBS respectively (GIBCO, Invitrogen Corporation), sodium pyruvate (1 mM), HEPES (10 mM), glucose (4.5 g/L), L-glutamine (2 mM), and penicillin and streptomycin (5000 IU/mL and 5000 μg/mL, respectively; Thermo Scientific). Insulin solution from bovine pancreas (0.01 mg/mL) was also added to OVCAR-3 medium and both JAr and OVCAR-3 medium was buffered with 1.5 g/L NaHCO_3_ (Pfizer). Cell lines were maintained in 75 cm^2^ sterile tissue culture flasks (NUNC, Thermo Fisher Scientific) at 37 °C in a humidified atmosphere with 5% CO_2_ and sub-cultured every 2–3 days as required. When the cells were 80% confluent they were either passaged or used in experiments. Viable cell numbers were determined using the trypan blue exclusion assay on a haemocytometer [54].

### 3.3. Combined Caspase 3/7, Membrane Integrity and Cell Viability Assays

The primary-derived granulosa cells, along with the KGN, JAr and OVCAR-3 cell lines (10,000 cells per well) were plated into sterile white (opaque) 96-well plates (Interpath) and clear sterile 96-well plates (Interpath) in 0.1 mL per well of complete cell culture medium for 24 h (granulosa, KGN and OVCAR-3 cells) and 2 h (for JAr cells) to allow cell adherence. Standard curves of 0–40,000 cells per well (primary granulosa cells) and 0–80,000 cells per well (for KGN, JAr and OVCAR-3 cells) in six replicate wells were plated into clear 96-well plates, alongside the test plates. After the initial cell adherence period, spent media (media deficient of nutrients and serum) were discarded and primary-derived granulosa, KGN, JAr and OVCAR-3 cells were treated with the two *D. orbita* fractions at concentrations of 0.005, 0.01, 0.05, 0.1, and 0.5 mg/mL in a final volume of 0.1 mL per well in triplicate wells, for 4 and 24 h at 37 °C + 5% CO_2_. 1 h prior to the end of eachincubation period two positive controls were added; one for apoptosis (1 μg/mL DNase I), and the second for necrosis (2 μL per well of a cell lysis buffer; Promega). This experiment was repeated on three separate occasions (*n* = 3).

#### 3.3.1. LDH Membrane Integrity and Caspase-Glo 3/7 Assay

After 4 and 24 h exposure to the *D. orbita* fractions in opaque 96-well plates, a combined membrane integrity and caspase 3/7 assay were performed on the primary-derived granulosa cells and KGN cells. The CytoTox-ONE Homogeneous Membrane Integrity Assay kit (Promega) was applied according to the manufacturer’s instructions. After the fluorescence was read at 535_EX_/590_EM_, the Caspase-Glo 3/7^®^ assay (Promega) was applied to the primary granulosa cells and KGN cells according to the manufacturer’s instructions for1h and the plates were read on full light to capture total luminescence. The LDH-membrane integrity assay alone was performed on the JAr and OVCAR-3 cells using the CytoTox-ONE Homogeneous Membrane Integrity Assay kit (Promega). This assay was repeated on three separate occasions (*n* = 3).

#### 3.3.2. Crystal Violet Cell Viability Assay

After 4 and 24 h exposure to *D. orbita* fractions in clear 96-well plates, media and all dead unattached cells were removed. The remaining adherent cells were rinsed with sterile 1× PBS and the crystal violet assay was performed [55]. The crystal violet assay is a colourmetric assay in which only the nuclei of live cells take up the crystal violet stain (0.5%) [55]. The absorbance in the wells of the crystal violet plates were read on an automatic spectrophotometer at 570 nm, with reference absorbance 630 nm using KC Junior Software. This assay was repeated on three separate occasions (*n* = 3).

#### 3.3.3. MTT Cell Viability Assay

After 4, 24, 48 and 72 h exposure to *D. orbita* fractions in clear 96-well plates, media and all dead unattached cells were removed. The remaining adherent cells were rinsed with sterile 1× PBS and the MTT assay was performed. The MTT assay measures the reduction of a yellow substrate to formazan, a purple precipitate, by the mitochondrial succinate dehydrogenase enzymes [56]. A solution of 0.5 mg/mL MTT (was added to wells in a final volume of 0.1 mL. The plates were then incubated for 1 h (JAr cells) and 18 h (KGN, OVCAR-3 and GC cells) at 37 °C and 5% CO_2_. At the end of the incubation period, 80 μL/well 20% SDS in 0.02 M HCl was added and plate was incubated for a further 1 h at room temperature in the dark. The absorbance was the measured at 570 nm, with reference absorbance 630 nm, using an automatic spectrophotometer using KC Junior software. This assay was repeated on three separate occasions (*n* = 3).

### 3.4. Detection of Apoptotic KGN and Primary-Derived Granulosa Cells by TUNEL

Primary-derived human granulosa cells and KGN cells were plated into Nunc Lab-Tek II CC2 Chamber Slides at 30,000 cells per chamber well in a final volume of 0.3 mL of DMEM/F12 + 10% FBS complete cell culture medium and incubated for 24 h at 37 °C + 5% CO_2_ to allow cell attachment to slides. Spent media (media deficient of nutrients and serum) and non-viable and non-adherent cellswere discarded and replaced with the *D. orbita* fractions at concentrations of 0.005, 0.01 and 0.05 mg/mL in a final volume of 0.3 mL of DMEM/F12 + 10% medium. A medium-only and a 1% DMSO control were also added before incubation for 4 and 24 h at 37 °C + 5% CO_2_. This experiment was repeated on three separate occasions (*n* = 3). The supernatant was removed and the cells rinsed with PBS before fixation with 4% paraformaldehyde for 25 min at room temperature. The DeadEnd™ Fluorometric TUNEL System (Promega) was performed as recommended by the manufacturer. Briefly the cells were treated with 0.2% Triton X-100 for 5 min, to permeablize the cells. The cells were rinsed in PBS before the addition of 50 μL of rTdT incubation buffer (Promega) at 37 °C for 1 h, protected from light. Cells were rinsed in 2× sodium chloride and sodium citratesolution (Promega) for 15 min at room temperature (25 °C) then rinsed again with PBS. The cells were counter-stained with 1 μg/mL propidium iodide (PI). A separate positive control slide of 1 μg/mL DNase I, and a negative control slide of buffer without rTdT, was prepared in conjunction with treatment slides. Cells were examined immediately and photographed at 200 and 400× magnification using a Fluorescence Olympus BX50 Microscope with filter Chroma 31001 at excitation 450–495 nm, Dichroic 505 and emission 515–555 nm for the green fluorescein of TUNEL and Chroma 31002 at excitation 515–550 nm, Dichroic 565 and emission 575–615 nm for the red fluorescence of the PI staining. Photomicrographs of four fields of view were taken for each treatment in each repeat of theassay and the number of TUNEL positive nuclei, stained bright green, were calculated as a fraction of the total number of PI stained nuclei, stained bright red, in each image. The average of the four fields was then calculated.

### 3.5. Statistical Analysis

All experiments were repeated on three independent occasions (*n* = 3) and the results are presented as mean ± 1 SEM. Two-way analyses of variance (ANOVA) using the sensitive contrast K Matrix analysis tests [57] were conducted to examine the effects of the semi-purified *D. orbita* fractions on caspase 3/7 activity, LDH release, cell viability and, to determine the number of apoptotic cells induced by the semi-purified *D. orbita* fractions using SPSS software package version 17. Homogeneity of variance was determined using the Levine’s Test of Equality of Error and the alpha value adjusted to <0.01 where homogeneity of variance was violated.

## 4. Conclusion

In conclusion, this study has identified the Australian marine mollusc *Dicathais orbita* as a source of potential anticancer agents with the potential to treat female reproductive cancers. The natural brominated muricid compounds tyrindoleninone and 6-bromoisatin selectively inhibit and kill reproductive cancer cell lines at low concentrations, while having minimal effect on primary-derived human granulosa cells. Furthermore, both tyrindoleninone and 6-bromoisatin induce cell death in the human granulosa cancer cell line KGN by apoptosis as opposed to necrosis at the lowest concentrations. Hence our data together with previous studies [30,32] suggests that these naturally occurring compounds are selective towards human cancer cell lines both *in vitro* and *in vivo* and thus are promising targets for the treatment of reproductive cancers.
